# Estimation of radiobiological parameters and repopulation dose equivalents using classical cell kinetics with hypotheses concerning cellular changes during radiotherapy

**DOI:** 10.3389/fonc.2026.1951206

**Published:** 2026-10-28

**Authors:** Bleddyn Jones

**Affiliations:** 1 Gray Laboratory, Oxford Institute for Radiation Oncology and Biology, Oxford, United Kingdom; 2 Green Templeton College, Oxford, United Kingdom

**Keywords:** cell kinetics, hypoxia, radiobiology, radiotherapy, repopulation dose equivalent

## Abstract

**Purpose:**

To study tumour responses and the changes in radiobiological parameters which may better inform decision making applied to overall time issues and treatment delays in a variety of histological tumour types during radiotherapy.

**Methods:**

Classical cell kinetic equations and radiobiological principles have been combined to study changes in effective doubling time (*T_eff_*) and repopulation dose equivalents (*Krepop*) during radiotherapy in a model which includes a non-proliferating radioresistant hypoxic fraction, reoxygenation, and a reducing cell loss factor (φ) governed by the tumour volume doubling time (*T_D_*), such that longer *T_D_* times will have longer apparent onset times of AR (accelerated repopulation).

**Results:**

The model found shortening of *T_eff_* at a time commensurate with the onset of accelerated repopulation in well-defined histological tumour types with their typical cell kinetic characteristics, along with diminishing extents of tumour hypoxia with time. The *Krepop* values show a steady increase during treatment and become closer to the constant *Krepop* values normally used in treatment interruption assessments for each histological class. Tumour classes with lower initial *φ* values show a greater persistence of hypoxia; those with very long initial *T_D_* values do not develop accelerated repopulation during or closely after radiotherapy, their *Krepop* values remaining low.

**Conclusions:**

The current use of constant *Krepop* values only after critical AR onset time durations may be sub-optimal in treatment interruption compensations since *Krepop* probably varies continuously with time due to progressive shortening of *T_eff_*. For shorter schedules, use of high *K* values (as used for longer schedules) could result in compensatory overdosage; lower compensatory doses obtained by using lower *Krepop* values should be preferred with no lag time to AR onset. Very prolonged interruptions probably require the highest *Krepop* values appropriate for the time at which compensation occurs.

## Introduction

The analysis of additional dose required to maintain isoeffective tumour control with increasing treatment duration in head and neck squamous cell cancers during radiotherapy in 1988 by Withers (1) did ultimately lead to BED (biological effective dose) modelling-based methods for the compensation of interruptions to treatments. BED is expressed as in Equation 1.

(1) BED=n×d(1+dαβ)

where *n* is the number of fractions, *d* the dose per fraction and *α/β* is the inverse of fraction sensitivity, the product *n*×*d* is the total dose. The usefulness of BED in Radiation Oncology is described elsewhere (2).

These are now well described, and used in clinical practice, although contain several pitfalls and often require compromises between maintaining acceptable tumour control and the available normal tissue tolerances (3). There is always some degree of uncertainty about the choice of radiobiological parameters used within BED based equations, which are to be preferred to the equivalent dose in 2 Gy fractions (EQD-2) method in some cases (4).

Whereas the normal tissue late effect α/β ratios are well-established and reasonably stable between patients, the tumour related parameters inevitably possess greater degrees of variation. The daily compensatory dose (in BED units) is given the symbol *Krepop*, which expresses the BED dose required per day to oppose repopulation. It is defined by Equation 2:

(2) $$Krepop=\frac{0.693}{\alpha \times T_{eff}},$$

where *α* is the intrinsic radiosensitivity and *T_eff_* is the relevant clonogenic doubling time (5, 6).

It has previously been assumed that there is an apparent time lag (*T_K_*) after which *K* operates, a probable oversimplification of the process called accelerated repopulation which occurs in some tumour types, but in this way is simple to apply (Fowler 1989) (6). However, *K* must be a variable which changes on a continuous basis, increasing rather slowly at first before it becomes sufficiently large to adversely influence tumour control. Following the suggestion of Fowler that the cell loss factor (*φ*) must probably reduce with time during radiotherapy, thereby reducing the effective doubling time of cells (*T_eff_*) towards shorter values which would be sufficient to explain the occurrence of accelerated repopulation. This hypothesis was taken further by Jones and Dale (7), who modelled a continuous change in *φ*, but the analytical solutions required integration of the exponential decline of *φ* to account for total repopulation and to simulate accelerated repopulation successfully. The resulting equations were somewhat daunting to apply.

The present manuscript presents an alternative way of using cell kinetic parameters to estimate *K* by using an iterative tumour control model which incorporates non-proliferative hypoxic cells and proliferative oxic cells with a flux of reoxygenated cells from the hypoxic to the oxic cellular compartment, a further development of that used by Scott (8).

Readers should be familiar with the essential principles and equations of cell and tissue kinetics. These are summarised in **Appendix A**. The model presented relies on the importance of the tumour volume doubling time (*T_D_*), which contains information on the cell cycle time, potential doubling time, and cell loss factor (as expressed in the final equation in **Appendix A**). Essentially, a *T_D_*is a summary of the natural history of its growth, and it will also determine the pattern of responsiveness to radiation therapy. For a tumour with a long *T_D_*, the cell loss factor is usually large, and it is reasonable to assume that it will take longer for such a tumour to reduce φ and achieve rapid effective repopulation (where a smaller value of φ is required) than will a tumour with a much shorter *T_D_*. This is analogous to the fact that a vehicle with a slow velocity will take a longer time to reach a fixed destination than another vehicle with a higher velocity.

## Methods

### Choice of parameters and mathematical software

The radiobiological parameters of the linear quadratic model are given with each graphic and are chosen to be illustrative for each histological tumour type, based on collections of data as in include Warenius and Britten etc (9), Deacon et al. (10) and Tubiana, Dutreix and Wambersie (11), the latter quoting α values of 0.24, 0.27, and 0.31 Gy^-1^, with α/β values of 8.3, 6.1 and 5.7 Gy for glioblastoma, squamous cell carcinoma (SCC) and adenocarcinoma respectively. These SCC related parameters can be regarded as low, and should be larger than those for most adenocarcinomas if in accordance with most clinical experience. In each case the radiosensitivity values were adjusted to give reasonable expectations for α/β and the achievable tumour control probability (*TCP)* at total doses using 2 Gy fractions, 5 treatments per week. For basal cell carcinomas the potential doubling time (*T_pot_*) values are around 8–9 days by Wright (12), and because of their long volume doubling times of often of a year or more, the cell loss factor is around 98%. Sarcomas and glial tumours (of mesodermal origin) will tend to have lower values of the cell loss factor than the carcinomas, based on experimental studies collated by Denekamp (see table 4-III, page 45) (13).

Programming used *Mathematica (Wolfram)* software, and the initial number of tumour clonogens (cells capable of repopulation) is set as 5×10^8^ in all examples, for comparative and tentative modelling studies which allows fair comparisons of the output changes, although it does not necessarily imply that tumour volume is the same at the onset of radiation since cellular and clonogenic density vary not only in different tumour histologies but even within such categories according to the tumour differentiation status and clearance rates of dead cells. The linear quadratic model of radiation effect is used throughout.

A list of cell kinetic and radiobiological parameter symbols and their definitions are provided in **Table 1**.

**Table 1:** Tabulation of parameter symbols, definitions and units.

Symbol	Definition	Units
*T_C_*	The cell cycle time	days
*GF*	The fraction of cells undergoing replication	fraction
*T_pot_*	The potential doubling time	days
*φ*	Cell loss factor which converts *T_pot_* to *T_D_*	%
*T_D_*	The tumour volume doubling time before commencement of treatment	
*T_eff_*	The effective tumour doubling time during treatment	
*k_B_*	The cell birth rate	new cells per cell/day
*k_L_*	The cell loss rate (which is hypothesised to become the flux of cells during reoxygenation)	lost cells per cell/day
*k_G_*	The growth rate constant	Net gain of new cells per cell/day
*z*	The rate parameter for change in cell loss with time	per day
α	The cell kill per unit dose	Gy^-1^
β	The cell kill per unit dose squared	Gy^-2^
N	Initial number of viable cells with clonogenic competency.	
*h*	The hypoxic fraction of cells	%
*f*	The mean inter-fraction time interval	days
*n*	The number of fractions	
*d*	The dose per fraction	Gy
*Krepop*	The dose equivalent per day required to oppose repopulation	Gy (in BED units)
TCP	Tumour control probability	%

### Description of model

The essential features, with radiobiological reasons, are as follows:

1. The tumour has proliferative oxic and non-proliferative hypoxic compartments, denoted by the hypoxic fraction (*h*), each of which have radiosensitivity modifications by their oxygen enhancement ratios, the α and β parameters reduced by factors of 1.75 (*q_α_*) and 3.5 (*q_β_*) respectively, which are probably ubiquitous and apply to all tumours, as described elsewhere (14). The number of cells in each compartment are *N_ox_* and *N_hyp_* and are counted after each fraction of radiotherapy.

2. The initial repopulation rate of the entire tumour (but occurring only in the oxic compartment) is described by the effective doubling time (*T_eff_*) which is the same as the tumour volume doubling time (*T_D_*) at the initiation of treatment. Thus *T_eff_* replaces *T_D_* during treatment since volume considerations are no longer appropriate during growth stasis or tumour shrinkage but tumour clonogens continue to proliferate.

3. After the first fraction of radiotherapy (and subsequently), the cell numbers in each compartment are counted, as is the new value of *h*. The mean inter-fraction interval (*f*, which is simply 7 days divided by *n_w_-1*, where *n_w_* is the number of fractions per week) is used to estimate the repopulation increment assuming exponential growth kinetics.

Initial simulations showed that the hypoxic fraction increased until a time that was approximated by three cell cycle (*T_C_*) durations (i.e. 3×*T_C_*), which probably reflects the delayed onset of cell killing. The same time interval was used to commence the initial reduction in *φ* from its initial value of φ_0_ to φ_t_ (at time *t*) by assuming exponential decay, as previously suggested (6). The time constant of exponential decay in *φ* (referred to as *z*) is chosen to be the exact inverse of the tumour doubling time at initiation of treatment, i.e. $\;z=\frac{1}{T_{D}}$. This was the original postulate and based on the known relationship between *φ*, *T_D_* and *T_pot_*, namely $\varphi =\left(1-\frac{T_{pot}}{T_{D}}\right)$, from which the inverse of *T_D_*(or z) is given by $z=\frac{1}{T_{D}}=\frac{\left(1-\varphi_{0}\right)}{T_{pot}}$ and which contains no 0.693 term. From characteristic Tpot values φ_0_ can be found. In the simulations typical human *T_C_* and *GF* values to provide characteristic *T_pot_* values which occur for specified histologies, and thence *z* is found and is later used to change the initial output of *T_eff_* (which is the *T_D_*) with increasing times.

The z parameter is used within a decreasing exponential equation, and can then have a relationship of 0.693/T_half_, the T_half_ then being the half time of the decay of cell loss which is entirely different from the doubling time of a tumour.

Now since the change in *φ* from a long value to a short value (and so to a shorter *T_D_*) should take much longer than from an initial moderate *φ* value associated with a moderate *T_D_*, and that this constant will be governed by the initial conditions which includes the histopathological structure of the tumour and the degree of diminution of its vascular supply in relation to tumour volume. Since *T_D_* (and thereby *T_eff_*) is dependent on *φ*, the change in *T_eff_* during radiotherapy will also depend on the same rate parameter. In this way tumours with long volume doubling times will take longer to develop accelerated repopulation (AR), which is consistent with present knowledge. For example, rapidly growing squamous cell carcinomas develop AR at between 21 and 28 days, before the 35–42 days required for transitional cell carcinomas and longer than 60 days for adenocarcinomas respectively (1, 15–17). It is also recognised in clinical practice that slow growing tumours normally show more delayed shrinkage responses, with perhaps a few exceptions where intra-mitotic radiation cell death does not predominate, as in lymphomas, leading to the idea that changes in *T_eff_* will change as some function of *T_D_*. Tumour shrinkage itself follows an exponential function of time and where continued shrinkage is observed sometimes years after radiotherapy (as in the clinical examples of low-grade gliomas and meningiomas), it suggests that the *T_eff_* time remains long so that the *φ* rate parameter, which applies to the change in *φ* must be small.

Reoxygenation process is well established in radiobiology, and appears to be important in influencing curability (18). A flux of cells is allowed to occur between the hypoxic and oxic compartments. The definition $\varphi =\frac{k_{L}}{k_{B}}$, the quotient of the cell loss and cell birth rates respectively, leads to Equation 3:

(3) $$k_{L}=k_{B}\varphi ,$$

and this cell loss rate is assumed to occur from the most hypoxic cells within the hypoxic compartment, containing the cells most likely to die over a 24 hr period, as would occur in growing conditions with the hypoxic fraction gradually increasing with time. During radiotherapy the flux of cells into the hypoxic compartment will reduce, and it is known that the reoxygenation process can commence rapidly in some high dose animal single fraction experiments since the hypoxic fraction immediately approaches 100% (19–21), but with conventional fractionated radiotherapy a delay is likely since the hypoxic fraction will inevitably continue to increase with the first few fractions. The onset of reoxygenation should then allow this vulnerable fraction of cells (represented by *k_L_*) to be ‘rescued’ each day, due to improving tumour oxygen partial pressures since many oxic cells will have died. This same proportion of cells in the hypoxic zone, situated in the better oxygenated zones of the hypoxic cell region, can be assumed to become sufficiently well-oxygenated to assume the radiobiological status of oxic cells. Consequently, this number of cells (*k_L_*× *N_hy_*_p_) is then removed from the hypoxic compartment and added to the oxic compartment each day, as in the assumptions made by Scott (8). Now *k_L_* can be expressed as the product *φ k_B_*, where *k_B_* is related to the potential doubling time (*T_pot_*) as *0.693/k_B_*, so that the to be as in Equation 4:

(4) $$k_{L}=\varphi_{0}\frac{0.693}{T_{pot}}.$$

4. The diminution of *φ* must then be reallocated in accordance with the elapsed time. The value of *T_pot_* is either taken from the literature for each histological tumour classification or from the quotient of the cell cycle time (*T_C_*) divided by the growth fraction. After the first few fractions, the growth fraction will increase so that *Tpot* will then approach *T_C_*. For daily fractions (five times per week) reoxygenation fluxes can be applied after each fraction, but if the inter-fraction interval exceeds 3 days, no reoxygenation is allowed after 3 days has elapsed (since successive applications of *k_L_* per day can within that time excessively deplete the hypoxic population, and it is known that reoxygenation after single fractions is rapid and ends within around 3–4 days in fast growing murine tumours (19–21). The parameter *k_L_* will inevitably reduce with time as influenced by the exponential reduction in φ. With inclusion of the time interval *f* between fractions, *k_L_*can be initially expressed as in Equation 5:

(5) $$k_{L}=f\frac{0.693\varphi_{0}}{T_{pot}}$$

for a duration of 3×*T_C_*, but thereafter as in Equation 6:

(6) $$k_{L}=f\frac{0.693\varphi_{0}e^{-z\left(t-3T_{C}\right)}}{T_{C}},$$

where *T_C_* is then preferred to *T_pot_* for the remainder of therapy for the reason explained above and $z=\frac{0.693}{T_{D}}$. The alternative use of *T_eff_* in this situation was found not to provide adequate reoxygenation in simulations.

The *T_eff_* time must shorten after the hypoxic fraction begins to reduce and will inevitably continue to shorten until the *T_pot_* value is obtained and further shorten to approach *T_C_* when the growth fraction should be very high, perhaps approaching 100%. This is consistent with some analyses of fractionation data, for example by Fowler and Harrari (22), where for head and neck cancer the effective doubling time (symbolised then as *T_p_*) approached 2 days, a reasonable assumption of the cell cycle time, with an α/β ratio of 8.6 Gy, based on head and neck cancer trial results published by Fu et al. (23). In the present study, *T_eff_* is initially kept constant as Teff being the same as the tumour volume doubling time as in *T_D_* or $T_{eff}=\frac{T_{pot}}{\left(1-\varphi_{0}\right)}$, until an interval of 3 cell cycle times, when the hypoxic fraction begins to fall, but is allowed to shorten due to the reduction in φ as governed by the rate constant (*z*), as in Equation 7:

(7) $$T_{eff}=\frac{T_{pot}}{\left(1-(\varphi_{0}e^{\left(-z\;(t-3T_{C}\right)}\right)}-\left(T_{pot}-T_{C}\right).$$

This equation allows *T_eff _* to approach *T_pot _* and then *T_C_* with time after a delay of 3×T_C_ which is subtracted from the time *t* in the exponent; the difference between *T_pot_* and *T_C_* is then subtracted to allow the function to approach an asymptote of *T_C_* when *t* is large.

5. Tumour cure probability is given by the Poissonian statistic: *e*^−^*^N^*, where N is the number of surviving cells in both oxic and hypoxic compartments.

6. The full details of the programming are given in **Appendix B**.

7. The outputs of total dose, *h, Teff, Krepop*, and *TCP* are plotted with increasing total treatment duration (days).

To assist the reader, **Tables 2**–**6** provide qualitative and quantitative summaries of the various processes and associated equations. It should also be noted that proliferative radiobiological responses are independent of dose since they essentially depend on the prevailing *T_D_* at the onset of radiotherapy. The hypoxia changes will depend on dose by influencing the surviving fractions in each compartment.

**Table 2:** A summary of the important tumour parameter changes during treatment.

Tumour dynamics during	
OXIC Compartment	Continues to repopulate at an overall rate given by Teff; reoxygenating cells are gained from the hypoxic compartment. Cell kill is greater than in hypoxic compartment
HYPOXIC Compartment	No repopulation but there is loss of cells to the OXIC compartment at rate of kL per day. Cell kill is smaller than in Oxic compartment.
T_eff_	This is initially T_D_, the tumour volume doubling time, but is progressively reduced after an interval of three cell cycle times by a change in φ governed by a rate constant hypothesised to be the inverse of T_D_.
k_L_	The loss rate of cells from the hypoxic compartment (during growth) reverses after treatment to be the reoxygenation rate to the oxic cells. This rate changes with φ.
Hypoxic fraction *(h)*	This changes according to the extent of cell killing in each compartment and influenced by the reoxygenation rate.

**Table 3:** Kinetic parameters during tumour growth (see **Appendix A** for further details).

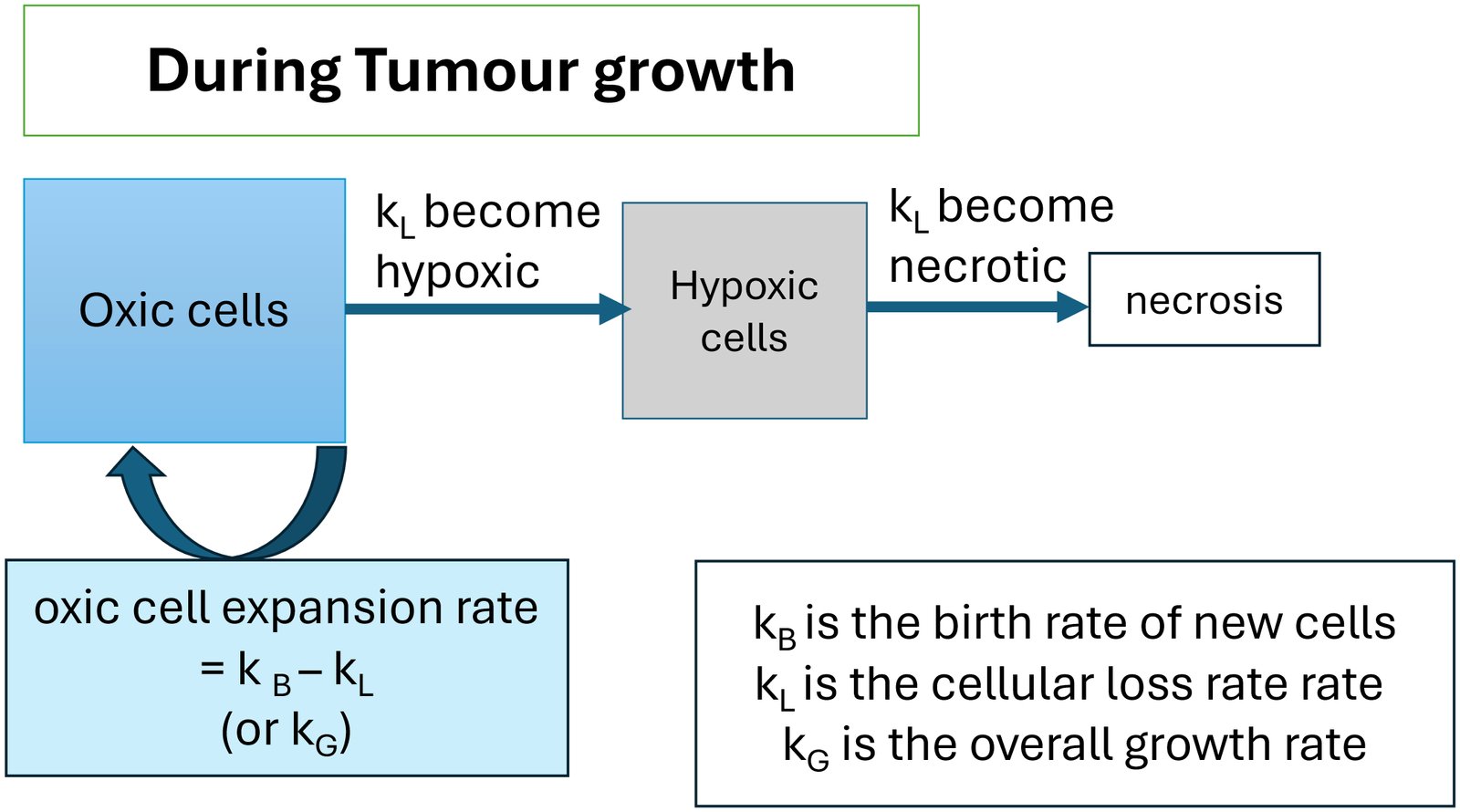

**Table 4:** Altered kinetic parameters during treatment, as hypothesised.

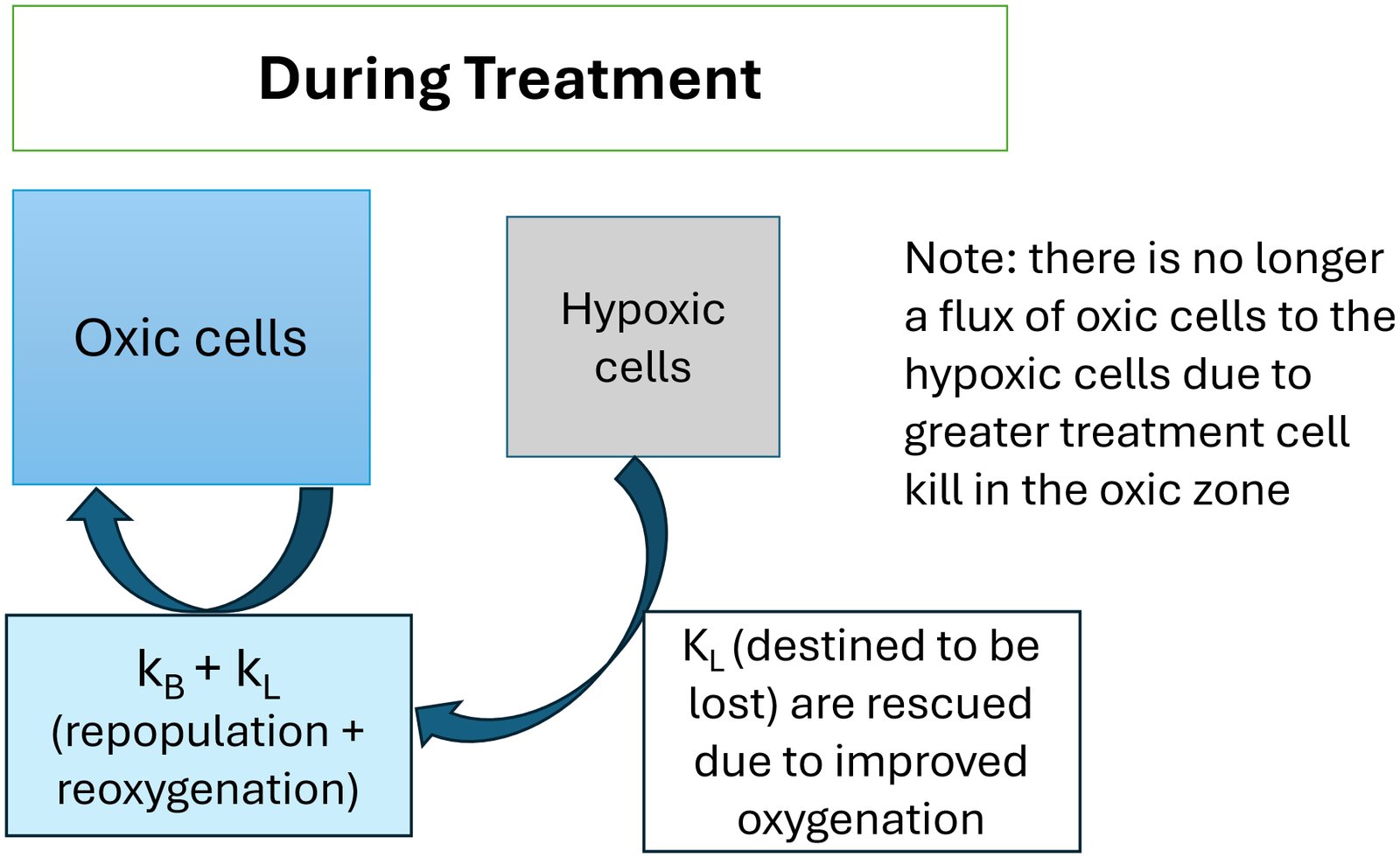

**Table 5:** Sequential stages in the computation process which are applied iteratively to each of *n* fractions of radiotherapy.

	Equation	Process
1	$f=\frac{\left(OTT\right)}{\left(n-1\right)}$	The inter-fraction interval is estimated from the expected overall treatment time (*OTT*).
2	*N (1-h)* cells are oxic*; N h* cells are hypoxic	Separation of the initial total of *N* cells into oxic and hypoxic compartments
3	$\text{Surviving fraction}\left(\text{SF}\right)=\frac{\alpha }{q_{\alpha }}d+\frac{\beta }{q_{\beta }}d^{2}$	Applied to the above numbers of oxic and hypoxic cells separately, using different q_α_ and q_β_ values
4	$z=\frac{1}{T_{D}}$	Estimating *z*, the rate parameter for *φ* from *T_D_*.
5	Initially, for 3 x TC days T_eff_ is the tumour volume doubling time, T_D_. but then changes to be: $T_{eff}=\frac{T_{pot}}{\left(1-(\;\varphi_{0}e^{\left(-z(t-3T_{C}\right)}\right)}-\left(T_{pot}-T_{C}\right)$	Oxic cell doubling times
6	New oxic cell number=Noxic SFoxic f Exp[0.693Teff]only for a time of 3 x *T_C_* days, reoxygenation provides	Repopulation applied only to surviving oxic cells over a time duration f (the mean inter-fraction interval)
7	Initial value: $k_{L}=f\frac{0.693\varphi_{0\;}}{T_{pot}}$, but after a time of 3 x *T_C_* days: $k_{L}=f\frac{0.693\varphi_{0\;}e^{-z\left(t-3T_{C}\right)}}{T_{C}}$	Estimation of *k_L_*
8	New Noxic=Noxic SFoxic f Exp[0.693Teff]+kL×Nhypox	Used for oxic cell population with reoxygenated cells added
9	$New\;N_{hypox}=N_{hypox}SF_{hypox}-k_{L}\times N_{hypox}$	Hypoxic compartment change due to reoxygenation
10	$New\;total\;number\;of\;cells=New\;N_{oxic}+New\;N_{hypox}$	Estimation of total cell numbers
11	$K=\frac{0.693}{\alpha \times T_{eff}}$	Estimated repopulation dose equivalent (*K*_repop_)
12	$h=\frac{New\;N_{hypox}}{New\;total\;number\;of\;cells}$	New hypoxic fraction (*h*)
13	$TCP=e^{-\left(new\;total\;number\;of\;cells\right)}$	Estimation of tumour control probability
14	***(n+m) (f-1)*** if an additional *m* fractions are given; any delays to treatment should be added to the accumulated time.	The actual overall treatment time if treatment interruptions have occurred and if additional fractions are given

**Table 6:** A qualitative summary of the trends observed, which are a consequence of the hypothetical assumptions made, their mathematical properties and influences as well as the ranges of parameters taken from the literature sources.

Symbol	Processes
*T_D_*	Determines initial *T_eff_*, and is related to the onset time of AR by controlling the rate of change in φ (parameter z) and ultimately *Krepop*.
*φ_0_*	Influences the reoxygenation rate by being directly related to *k_L;_*also influences initial *T_D_*.
*h*	Influences TCP depending on its persistence.
*α* and *β*	Influences TCP.

It should be noted that *T_D_*, *T_pot_* and *h* are measurable parameters, although the last two are not routinely available. The increments in *Krepop* lead from knowing *T_D_* and the likely or estimated *φ_0_****,***and is independent of the other parameters apart from *α*.

AR, accelerated repopulation.

## Results

The following graphics show the radiobiological outputs, for increasing overall treatment durations of fractionated radiotherapy, applied to different histological classes of cancers, with their typical cell kinetic parameters. The y-axis is labelled for percentages or the units of each of the responses, which are coded by the colour-code scheme and with additional labelling text nearby each curve. Readers should note that the scale also applies to the total dose (in units of Gy), the T*eff* time (in days), and the *Krepop* value in Gy day^-1^ multiplied by 100, and which is labelled as *Krepop* for ease of identification: for example, *K* = 20 on the scale is the same as 0.2 Gy day^-1^.

**Figure 1** shows the estimated responses for squamous cell carcinomas (SCC) with relatively short volume doubling times (*T_D_*) of around 38 days with a high tumour control probability with total doses between 60 and 80 Gy when given in five fractions per week. T_eff_ reduces efficiently to allow *Krepop* to increase in a time frame which is compatible with the analysis of Withers (1), where the initial and final doubling times (around 70 days later) were estimated to be around 40 and 4 days respectively, the latter value close to published estimates of median *T_pot_* values (24), and even shorter times found by Fowler and Harrari (20), who analysed further fractionation studies which suggested shorter doubling times occurring with extension of treatment duration. The hypoxic fraction increases initially before a progressive reduction occurs and *T_eff_* begins to reduce towards *T_pot_* and ultimately to approach *T_C_*. The resulting steep TCP dose response curve is steep, but illustrate the probabilistic response of an individual cancer rather than the shallower TCP curves obtained for a population of heterogenous tumours which contain different initial clonogen numbers and a statistical spread of the radiobiological parameters. The shortening of *T_eff_* down to values of 7–5 days where accelerated repopulation would manifest between 20 and 30 days, as expected for SCC, as also shown by the steadily increasing *Krepop* at that time (0.3 Gy day^-1^ and increasing) and which then approaches published values, e.g. 0.6-0.9 Gy day^-1^ (1, 22) at longer times.

**Figure 1:**
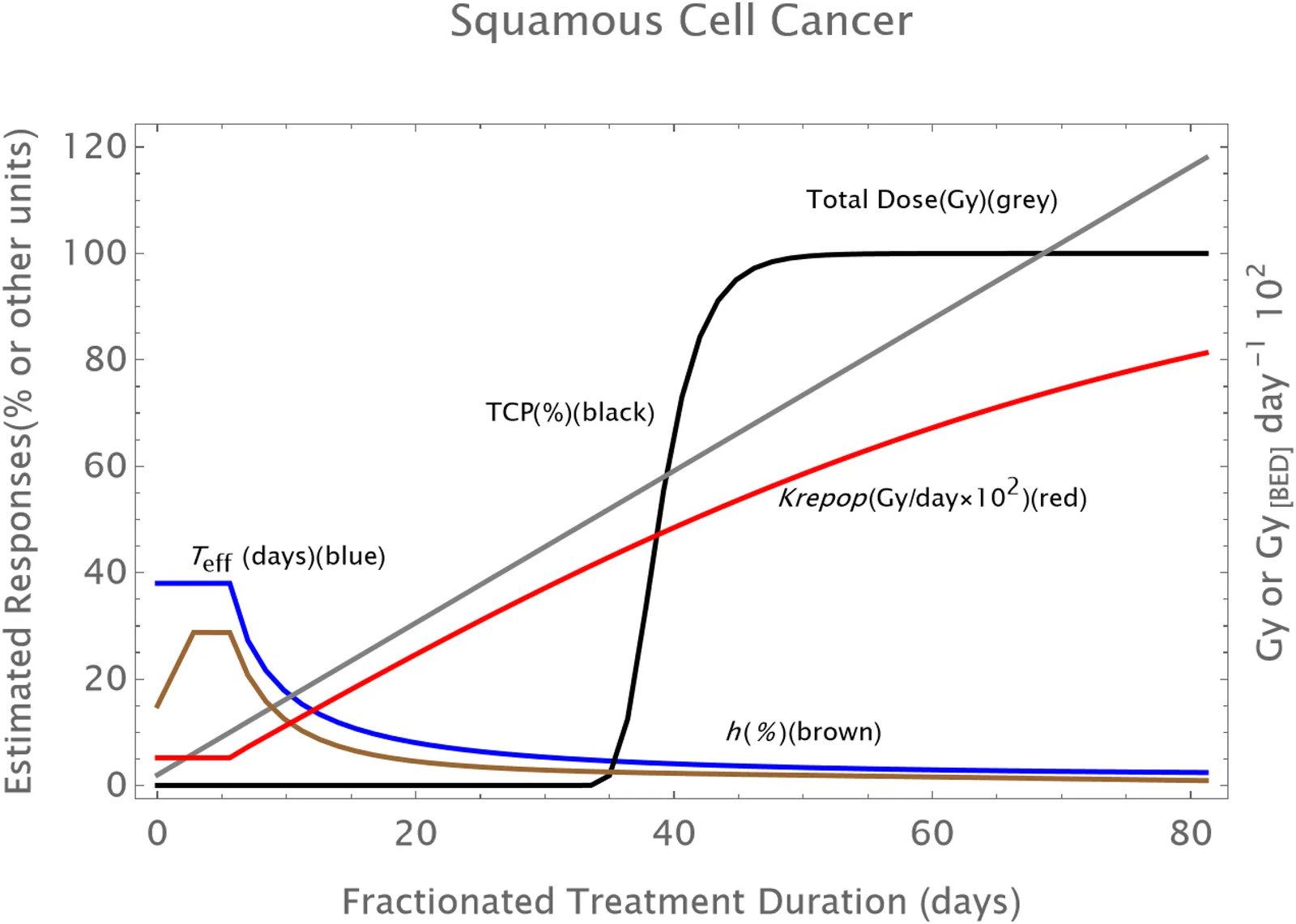
Simulation of a squamous cell carcinoma treated by radiotherapy using 2 Gy fractions five times per week. Assumed parameters: *T_C_* = 1.9 days, *φ_0_*= 90%, *T_po_*_t_=3.8 days, α_L_=0.36 Gy^-1^; β_L_=0.036 Gy^-2^, *h* = 15%. Each curve or line is labelled and given separate colours: Total dose is grey in units of Gy, TCP is black (with probability expressed as percentages), T_eff_ is blue (units of days) and starts at the *T_D_* value, the hypoxic fraction *h* is brown (expressed as percentages) and the estimated *Krepop* values are red (in units of Gy day^-1^ ×100 in order to be readable), so should be divided by 100 to give the correct units of Gy day^-1^.

**Figures 2a, b** simulates responses for two adenocarcinomas. The faster growing example with *T_D_* = 75 days, where *T_eff_* values shorten to cause significant increments in *Krepop* at around 40–50 days and onwards, again to values typical for some adenocarcinomas of around 0.3 Gy day^-1^ at the longest duration. The slow growing adenocarcinoma, with a *T_D_* of over 270 days, does not show any significant increment in *K* over 70 days, with *T_eff_* values remaining above 50 days after 70 days treatment duration.

**Figure 2:**
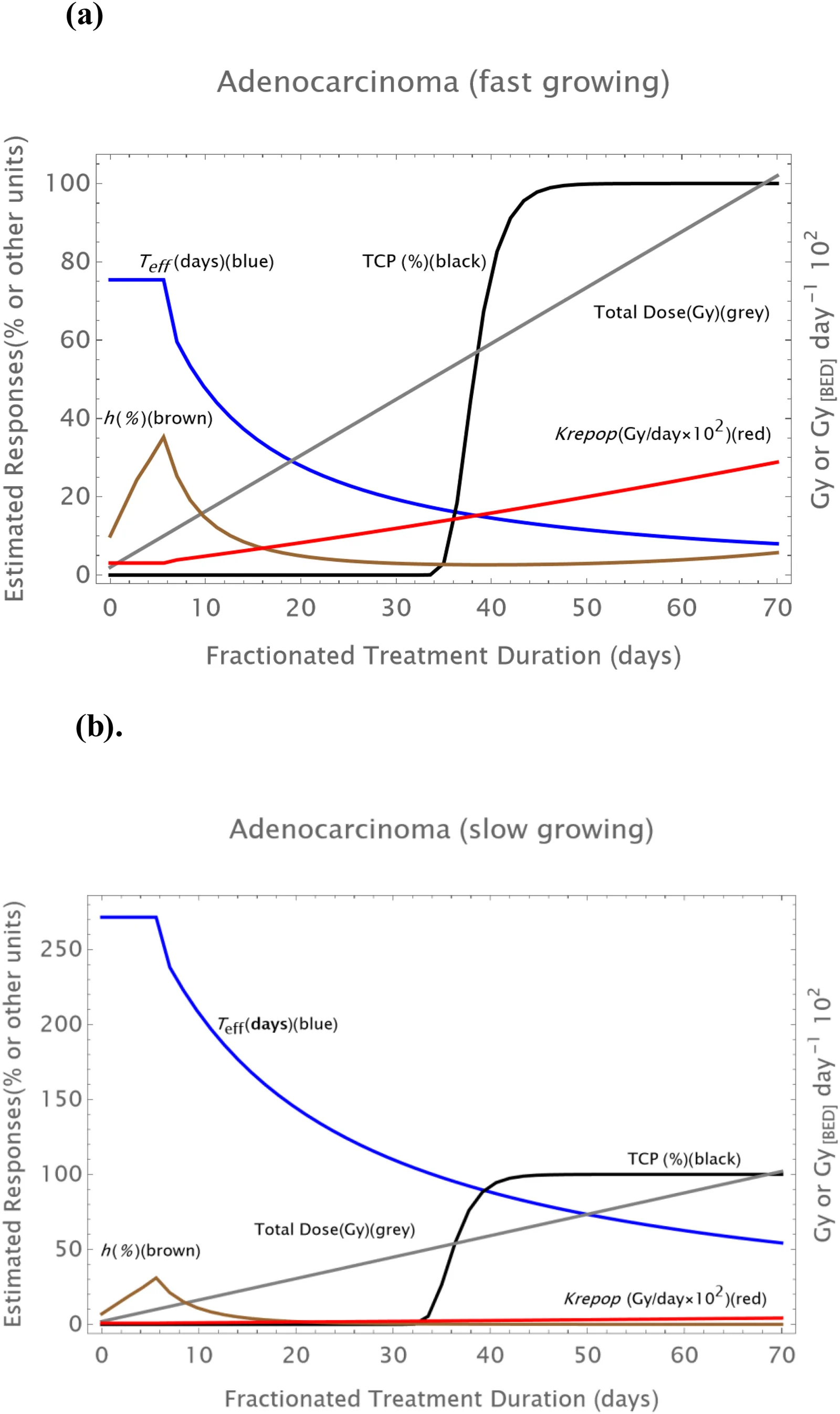
**(a, b)** Simulations of two adenocarcinomas treated by radiotherapy using 2 Gy fractions five times per week for **(a)** a relatively fast growing example with parameters: *T_C_* = 1.9 days, *φ_0_*= 86%, *T_po_*_t_=10.6 days, α_L_=0.3 Gy^-1^; β_L_=0.05 Gy^-2^, *h* = 10%. and **(b)** a slower growing example with parameters *T_C_* = 1.9 days, *φ_0_*= 93%, *T_po_*_t_=19 days, α_L_=0.3 Gy^-1^; β_L_=0.05 Gy^-2^, *h* = 7%. Each curve or line is labelled and given separate colours: Total dose is grey in units of Gy, TCP is black (with probability expressed as percentages), T_eff_ is blue (units of days) and starts at the *T_D_* value, the hypoxic fraction *h* is brown (expressed as percentages) and the estimated *Krepop* values are red (in units of Gy day^-1^ ×100 in order to be readable), so should be divided by 100 to give the correct units of Gy day^-1^.

**Figure 3** shows the estimated responses for a slow growing basal cell carcinoma with a high cell loss factor (98%), causing a long *T_D_* and *T_eff_*. The low initial *Krepop* values do not appear to increase significantly over the course of the study and are around 0.03 Gy day^-1^ at 30 days to 0.05 Gy day^-1^ at 55 days. Even at 70 days the T_eff_ is long at around 50 days, having reduced from an initial value of over 350 days.

**Figure 3:**
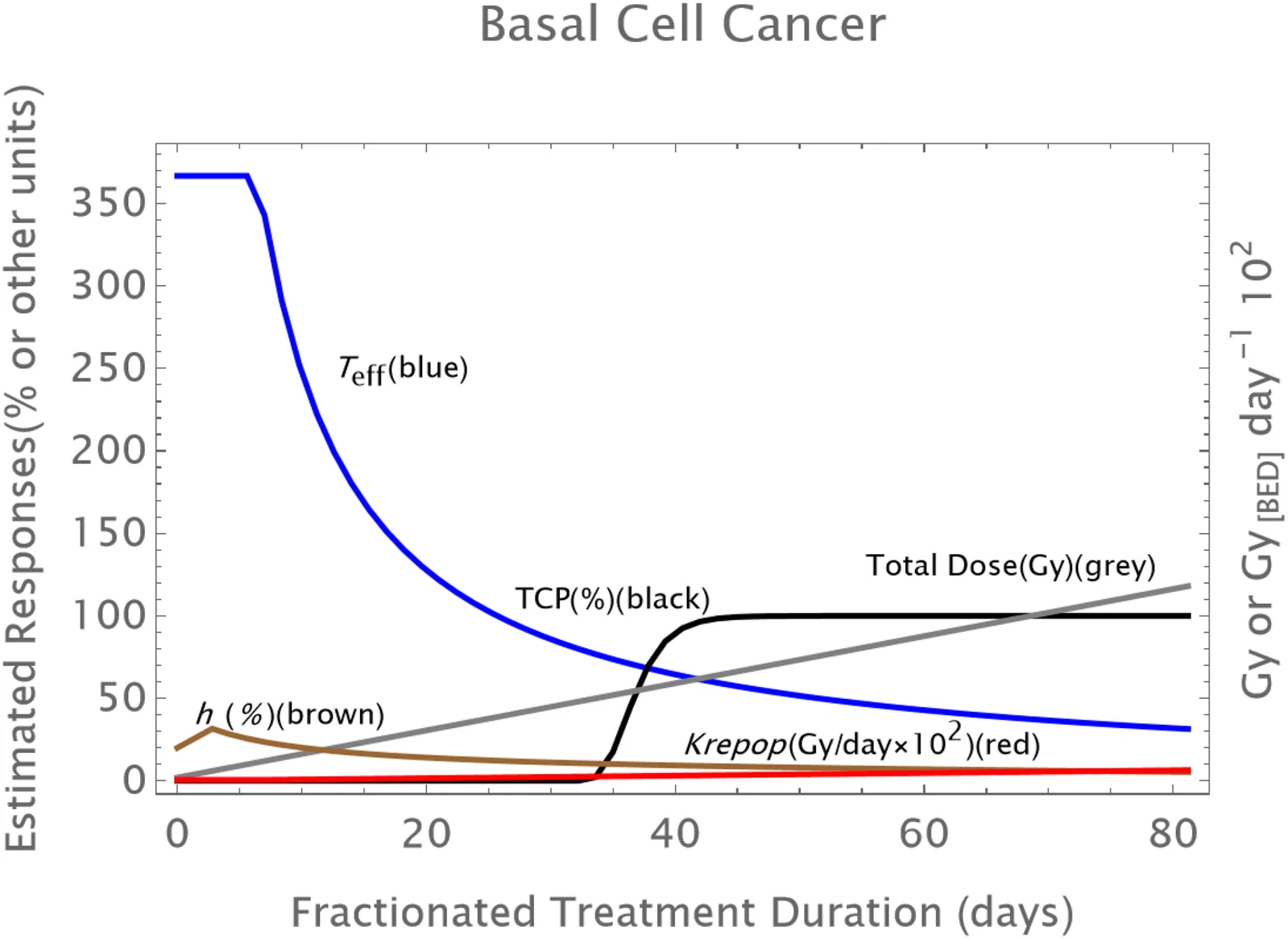
Simulation of a basal cell carcinoma treated by radiotherapy using 2 Gy fractions five times per week. Assumed parameters: *T_C_* = 2.2 days, *φ_0_*= 98%, *T_po_*_t_=7.3 days, α_L_=0.34 Gy^-1^; β_L_=0.04 Gy^-2^, q_α_=1.75;q_β_=3.25; *h* = 20%. Each curve or line is labelled and given separate colours: Total dose is grey in units of Gy, TCP is black (with probability expressed as percentages), T_eff_ is blue (units of days) and starts at the *T_D_* value, the hypoxic fraction *h* is brown (expressed as percentages) and the estimated *Krepop* values are red (in units of Gy day^-1^ ×100 in order to be readable), so should be divided by 100 to give the correct units of Gy day^-1^.

**Figure 4** refers to a glioblastoma multiforme, typically rapidly growing but with a *T_pot_* of 10 days, as suggested by Steel (25), which implies a growth fraction of around 20% if *T_C_* is around 2 days. The glial classification suggests a mesodermal origin with a potentially low *φ*, which consequently can allow a volume doubling time, in this example, of around 30 days which is typical of clinical practice: the estimated *φ_0_* is then around 66%. The directly measured hypoxia is substantial (26) and is estimated to persist on the graphic at a significant proportion during radiotherapy, as has been suspected by other studies (27). This would be a consequence of the lower initial φ in the present model.

**Figure 4:**
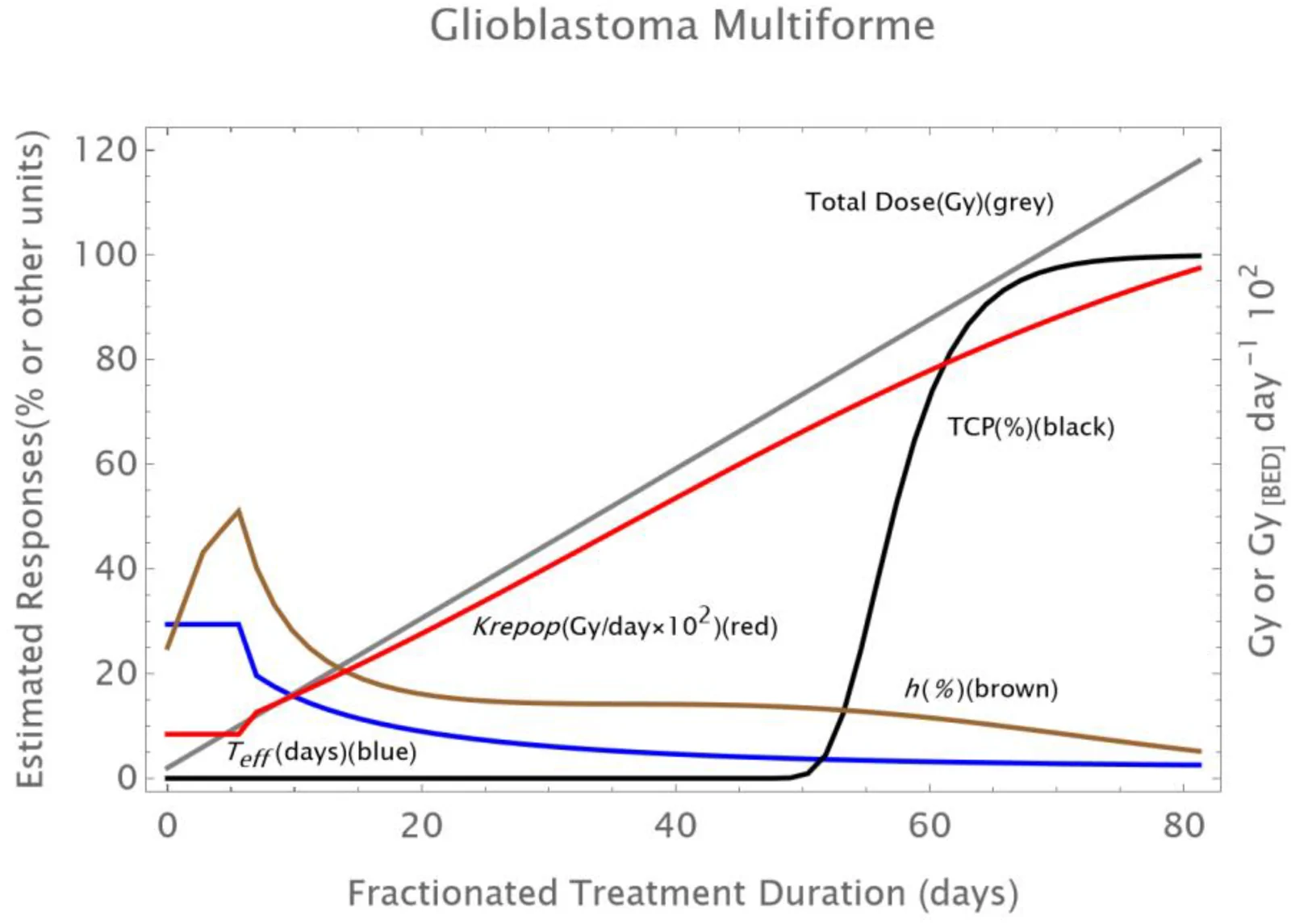
Simulation of a glioblastoma multiforme treated by radiotherapy using 2 Gy fractions five times per week. Assumed parameters: *T_C_* = 2 days, *φ_0_*= 66%, *T_po_*_t_=10 days, α_L_=0.28 Gy^-1^; β_L_=0.03 Gy^-2^, *h* = 25%. Each curve or line is labelled and given separate colours: Total dose is grey in units of Gy, TCP is black (with probability expressed as percentages), T_eff_ is blue (units of days) and starts at the *T_D_* value, the hypoxic fraction *h* is brown (expressed as percentages) and the estimated *Krepop* values are red (in units of Gy day^-1^ ×100 in order to be readable), so should be divided by 100 to give the correct units of Gy day^-1^.

**Figures 5a, b** consider examples of a slow- and fast-growing sarcomas, where φ can be lower due to the mesodermal tissue origin, although in these examples not as low as in some experimental animal sarcomas. There appears to be reduction of hypoxia with time but with persistence at a lower but significant level. The *Krepop* values become significant during radiotherapy in the fast-growing example, but not in the slow growing sarcoma.

**Figure 5:**
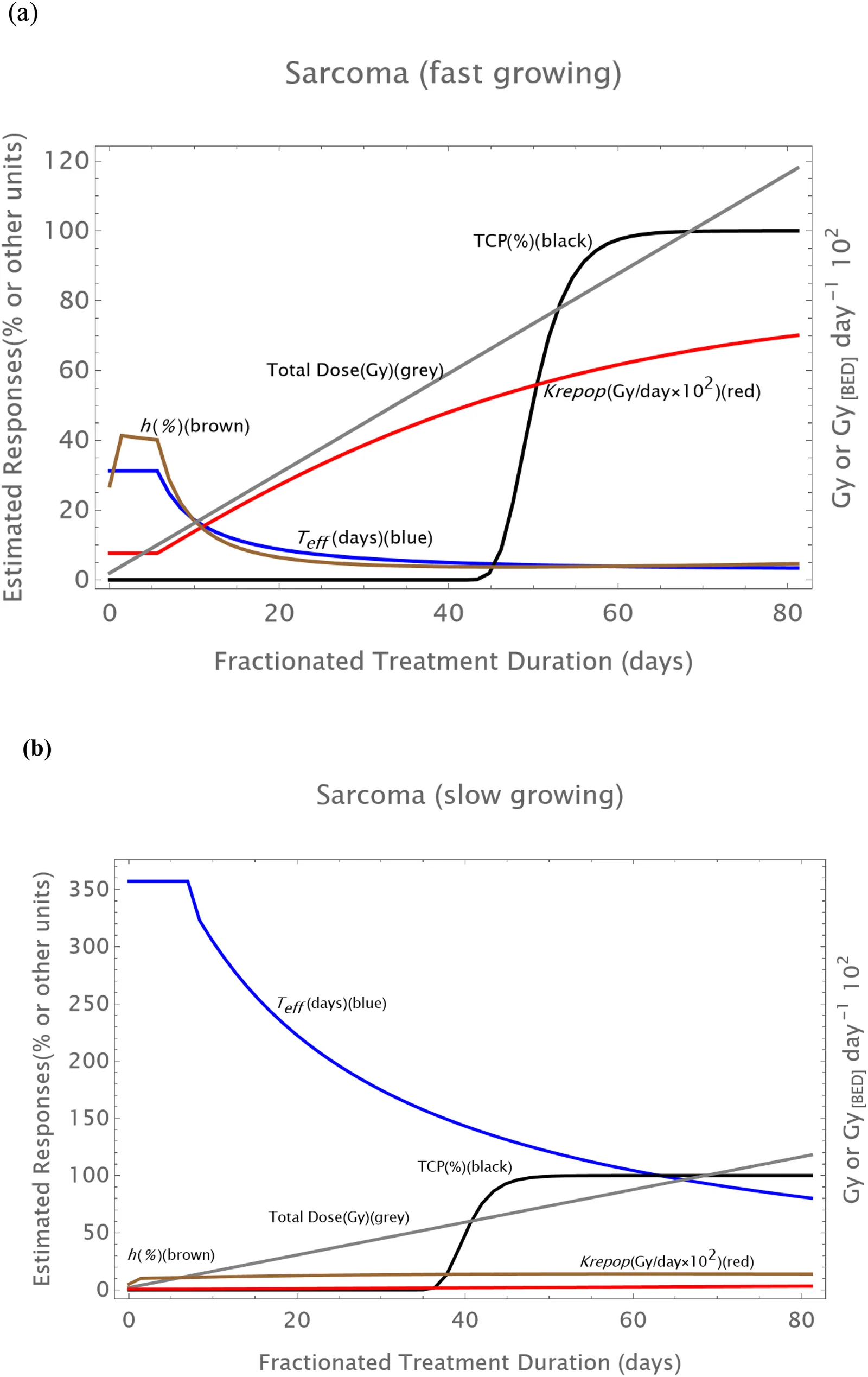
**(a, b)** Simulation of respective fast- and slow-growing sarcomas treated by radiotherapy using 2 Gy fractions five times per week. The assumed parameters are: **(a)** *T_C_* = 2 days, *φ_0_*= 85%, *T_po_*_t_=5 days, α_L_=0.29 Gy^-1^; β_L_=0.03 Gy^-2^, *h* = 27%; **(b)** *T_C_* = 2.5 days, *φ_0_*= 93%, *T_po_*_t_=25 days, α_L_=0.26 Gy^-1^; β_L_=0.05 Gy^-2^, *h* = 5%. Each curve or line is labelled and given separate colours: Total dose is grey in units of Gy, TCP is black (with probability expressed as percentages), T_eff_ is blue (units of days) and starts at the *T_D_* value, the hypoxic fraction *h* is brown (expressed as percentages) and the estimated *Krepop* values are red (in units of Gy day^-1^ ×100 in order to be readable), so should be divided by 100 to give the correct units of Gy day^-1^.

**Figure 6** is the only example of hypofractionated treatment shown, but which shows a marked increase in TCP towards the end of a 49 Gy course (7 fractions, three per week). The hypoxia reduces sharply around day 6, Teff does reduce during treatment but the *Krepop*, although increasing to 0.16 Gy per day, remains low at 14 days.

**Figure 6:**
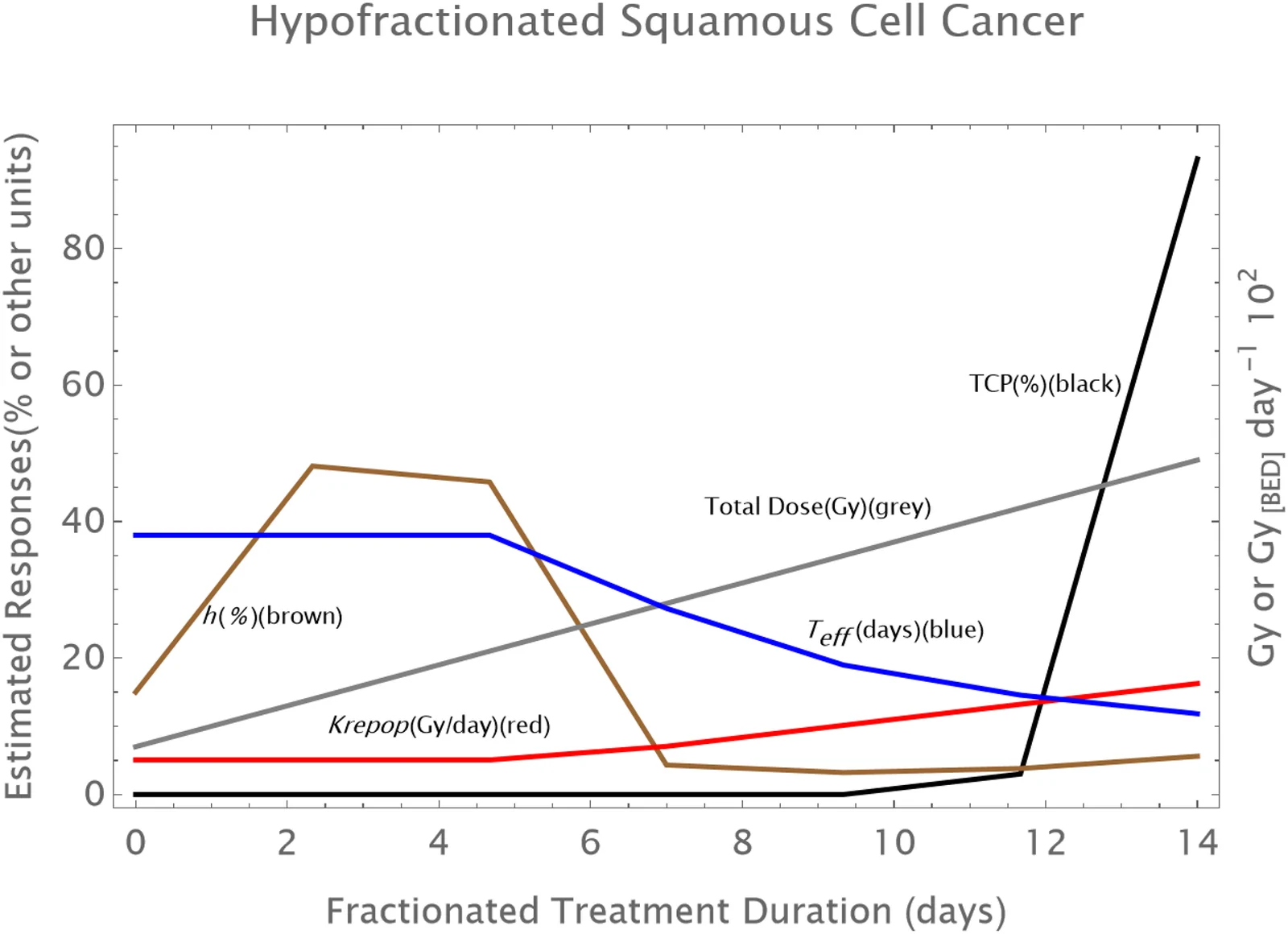
Simulation of a squamous cell carcinoma treated by radiotherapy using 7. Gy three times per week. Assumed parameters: *T_C_* = 1.9 days, *φ_0_*= 90%, *T_po_*_t_=3.8 days, α_L_=0.36 Gy^-1^; β_L_=0.036 Gy^-2^, *h* = 15%. Each curve or line is labelled and given separate colours: Total dose is grey in units of Gy, TCP is black (with probability expressed as percentages), T_eff_ is blue (units of days) and starts at the *T_D_* value, the hypoxic fraction *h* is brown (expressed as percentages) and the estimated *Krepop* values are red (in units of Gy day^-1^ ×100 in order to be readable), so should be divided by 100 to give the correct units of Gy day^-1^.

In **Figure 7** plots of *Krepop* with treatment duration are shown for a wide range of *T_D_* values, indicating the importance of this parameter. *T_D_* values of 20 and 130 days are respectively predicted to have *Krepop* values of 1.1 and 0.1 Gy day^-1^ at 70 days.

**Figure 7:**
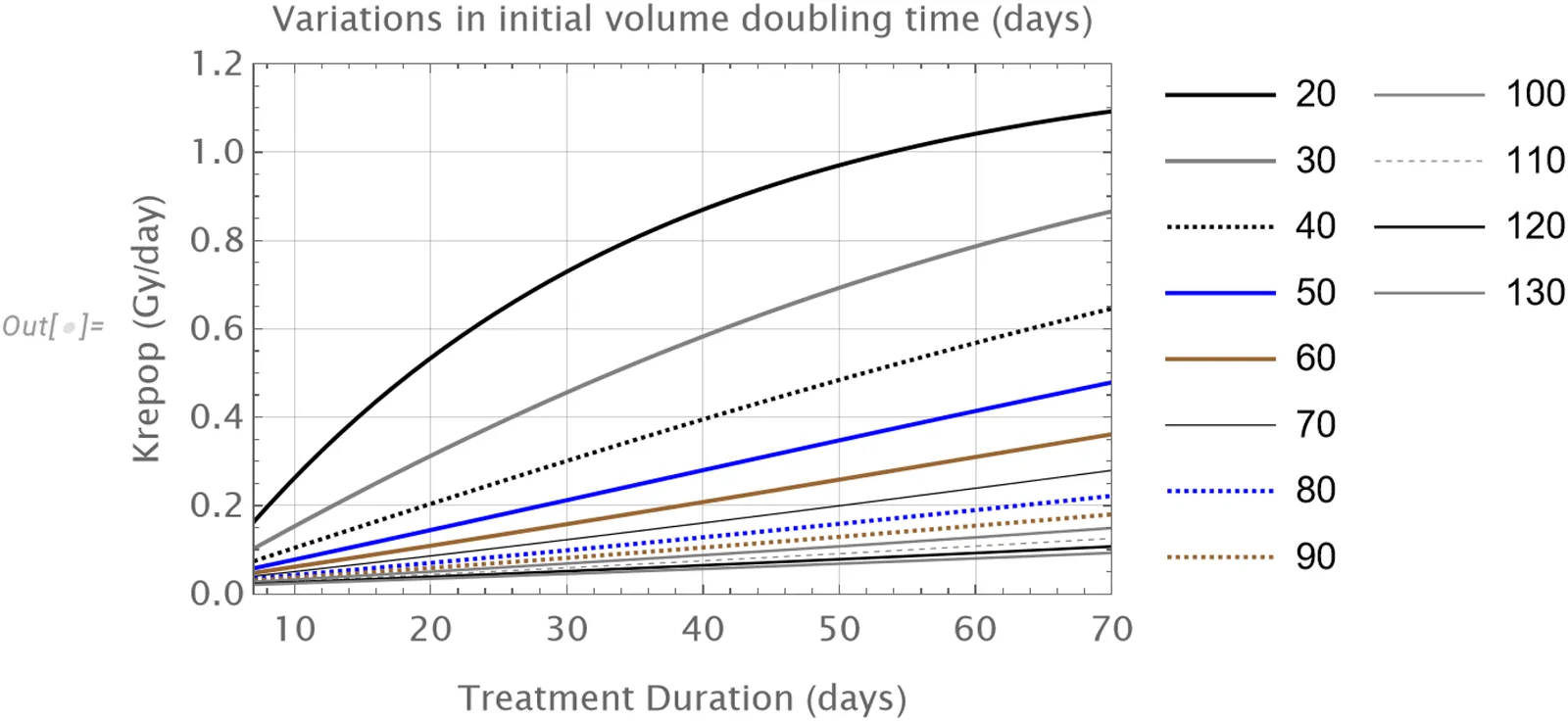
Plots of K (repopulation dose equivalent) values with treatment duration for tumours and variation of tumour pre-treatment volume doubling times (*T_D_*), as indicated by values in units of days. The initial conditions are *T_C_* = 2 days, *φ_0_*= 90% and α=0.3 Gy^-1^. These results are not dependent on the dose fractionation schedule.

A 3-D plot of estimated *Krepop* values with variations in φ_0_ and T_D_ is shown in **Figure 8**, whilst maintaining a constant α radiosensitivity parameter. Some of the extreme values are unrealistic but most are relevant and demonstrate the sensitivity of the initial condition parameters.

**Figure 8:**
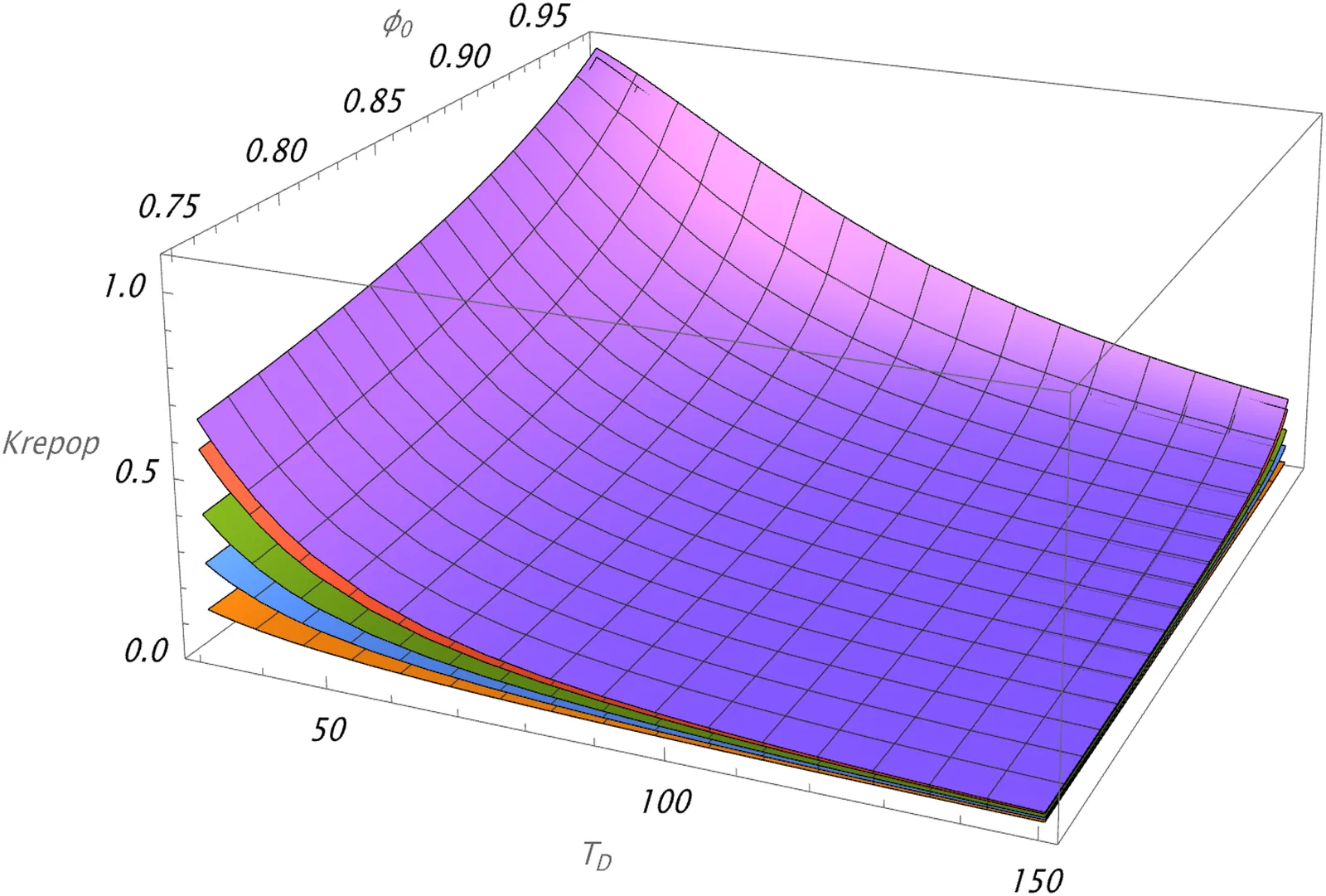
A 3-D plot of *Krepop*, the repopulation dose equivalent in units of Gy day^-1^) at treatment durations of 10 (brown), 25 (blue), 40, (green), 60 (orange) and 70 (purple) days after initiation of radiotherapy, with variation in the initial conditions *φ_0_* and *T_D_*, which shows the sensitivity to these parameters. Not all the phase space shown is applicable since, for example, tumours with the highest values of *φ_0_*will normally have the longest *T_D_*values and not the shortest. These results are not dependent on the dose fractionation schedule.

The above graphics mostly considered the conventional use of five treatment fractions per week. Increasing the *f* value can be used to estimate all of the above the radiobiological responses for more hypo-fractionated treatments giving 7 Gy per fraction, 2 or 3 times per week (as in **Figure 6**), so that similar graphical displays can be achieved, but at shorter durations, so further such graphical outputs have not been included in the present manuscript. For the *Krepop* values, however, it is only necessary to assume the same changes in *T_eff_* with time as already given for the conventional fractionation (2 Gy, five times per week), as are given in **Figures 1**–**4**, since *T_eff_* changes are not necessarily influenced by the fractionation pattern, but by the initial condition *T_D_* and *φ* values.

The functional contributions of each initial radiobiological parameter are summarised in **Table 5**. The modelling suggests that *T_D_* and *φ_0_* are important. A long *T_D_* is followed by delayed AR, but a short *T_D_* by a more rapid onset of AR. A high φ_0,_ (e.g. ≥ 90%) will promote rapid reoxygenation but if low (e.g. ≤ 85%) will allow greater persistence of hypoxia, although in each case modified by T_D_, which determines the rate of change in *φ*. The TCP results, in general, are typical for the tumour types under consideration and they all approach 100% at different total doses which, in some cases, would exceed normal tissue tolerances and are unachievable in clinical practice unless some additional risk is taken by using special techniques such as brachytherapy or other highly focussed beams.

## Discussion

The arguments presented are all consistent with known cell kinetic theory and the model contains relevant radiobiological processes. It is noted that *T_D_*, the volume doubling time was hypothesised to determine the rate parameter *z* for changes in *φ* and by doing so the reductions in *T_eff_* with increasing time, and that *φ_0_***(**which partly determines *T_D_*) influences the reoxygenation rate. Consequently, *T_D_* is hypothesised to be the observed parameter which probably best reflects changes in *φ* and *T_eff_.* Other consequences do emerge algebraically within the various equations, but are based on the above assumption, for example on the onset of effective AR, and the repopulation dose equivalent results. The simulations, although based on the chosen parameters are illustrative of different histological tumour types, are compatible with literature-based outcomes as noted in the results section for each graphic.

The overall model has important limitations which include dependency on the linear quadratic model of radiation effect, fixed oxygen modification factors, absence of patient heterogeneity (and so applies only to individual cases), a completely non proliferating hypoxic compartment but which does contribute to reoxygenation and so the proliferating pool of cells, the use of a fixed clonogen number (for purposes of comparative TCP values), and a lack of explicit vascular dynamics although the rate of change of *φ* and the reduction in *T_eff_*are probable consequences of such changes. Parameter uncertainty is another major issue, but which further research might improve.

Some previous clinical studies, although containing large numbers of patients, failed to show satisfactory tumour outcome correlations, especially with *T_pot_* and *SF_2_* (the surviving fraction after 2 Gy, a measure of radiosensitivity at low dose per fraction) (28–31). This was not surprising since the information provided by these two parameters is incomplete and does not include tumour volume doubling times, hypoxic fractions and reoxygenation in a more comprehensive model. Also, *T_pot_* and *SF_2_* will tend to oppose each other since *T_pot_* is expected to be lowest in tumours where radiosensitivity is highest. At later times, researchers sought to identify proliferative markers from microarrays, as in the work of Starmans et al. (2008), but again proliferation capability alone is only a single factor in the process of obtaining successful radiotherapy tumour cure; reoxygenation and cell loss related processes are also necessary for unmasking proliferation (32).

Despite the considerable enthusiasm and efforts made, *T_pot_* cannot be a good indicator of prognosis since, for example, the *T_pot_* of a highly curable basal cell carcinoma (BCC) and mostly incurable glioblastoma are probably similar, but their *T_D_* values vary significantly by around a factor of 10 due to the substantially higher *φ_0_* in the former tumour. Such results undoubtedly encouraged a diminishing respect for the established science of cell kinetics, especially in clinicians and other scientists with a more limited understanding of the subject. It is time to re-evaluate this standpoint, especially if the use of specific genetic mutations can correlate well with the relevant cell kinetic and radiobiological parameters so that such a cell-kinetic based model can be better informed. It may be possible that retrospective data could be used to reanalyse these studies if information on likely *T_D_* values are available, e.g. mutations in the hedgehog signalling pathway is characteristic of BCCs (33). This pathway controls proliferation, differentiation and, perhaps even more importantly, the fate of tumour cells. In the present time, many cancer patients have had multiple pre-treatment imaging investigations, so that *T_D_*estimates can be made, so that from typical *T_pot_* values the *φ_0_*can be estimated.

Another consideration is the variation in tumour cell kinetics with distance from a tumour blood vessel. Tannock (34) used tritiated thymidine to show that the estimated *T_pot_* values and growth fraction (*GF*) reduce with increasing distance from a blood vessel towards the location of tumour necrosis, although the use of such a radiation-based technique could perturb the findings. A similar result was obtained using the stathmokinetic method (using Vincristine) by Jones and Camplejohn (35), which estimated cell birth rates (inversely related to *T_pot_*) with distance from a blood vessel to necrosis but involved no radiation exposure. These experiments suggested the existence of a progressive reduction in proliferation, due to diminishing amounts of oxygen and essential nutrients, towards a hypoxic zone where cells do not repopulate although appearing to be viable but will have a greater probability of cell death to form necrotic debris. The gradient in *T_pot_*and GF reduction is also important since this implies that the apparent overall doubling time of all cells will be much longer and approach *T_D_*. Also, it is probable that the gradient of proliferation will be reduced in favour of shorter doubling times in a shrinking tumour, allowing T_eff_ to eventually approach *T_pot_* and *T_C_*. The initial *k_L_* values obtained imply that the half-time of cell death (due to hypoxia and nutrient lack) will be of the order of several days. For example, in **Figure 1** (squamous cell carcinoma) the first *k_L_*is given by $\varphi_{0}\frac{0.693}{T_{pot}}$ or 0.9×0.693/3.8, which is 0.164 per day, which suggests a half time of tumour cell loss (*T_loss_*), due to oxygen and nutrient depletion, of around 0.693/0.164 = 4.2 days, whereas at 80 days after commencement of radiotherapy *k_L_* reduces to 0.063 per day, and the half-time of tumour cell death is then estimated to be around 11 days (probably in well oxygenated conditions). It may be commented that these half-times appear long but this is probably due to the adaptation of malignant cells to hypoxic conditions by using anaerobic respiration (the Warburg effect) and other metabolic pathways to provide energy (36).

The importance of the initial tumour volume doubling time (*T_D_*) has been used to determine the parameter *z* (or rate of change in φ) in the model. The values obtained are lower than those found to simulate accelerated repopulation best in a model which did not include the effect of hypoxia on the tumour cure dose and which consequently used higher α values (7).

The new model results have suggested several principles concerning the initial delay of responses and the more graduated changes in proliferation with time than has been assumed with modelling techniques where the onset of accelerated repopulation occurs as a sudden discontinuity. The results suggest that consideration should be made of using a variable *Krepop* value according to the time at which treatment interruptions corrections are made. Where relevant, the final week or days of treatment should be used as being the relevant time to assess a *Krepop* value. Also, the use of more hypo-fractionated treatments does not necessarily imply that repopulation effects can be ignored, but that lower *Krepop* values might be used to compensate for significant treatment interruptions.

The α parameter used in the estimation of *Krepop* assumes is normally considered to be a constant value, the intrinsic value without modification by hypoxia etc, and its use in the definition of *Krepop* was based on compatibility with the BED concept and the Gy per day units of *Krepop* are essentially in BED (5, 37, 38), a fact which is often misunderstood.

Should *α* vary with time? The *T_eff_* value will vary with time and both *α* and *T_eff_* form the denominator in the equation which defines *Krepop*. Each tend to oppose each other in that if α increases due to more rapid proliferation Teff will be reducing, so that to some extent the net change in *Krepop* is reduced. However, the α values which are used are derived from *in vitro* experiments where cells have optimum growth conditions, so that the published values represent rapidly growing tumour cells for each histological class. It seems, therefore, that these α values are reasonable for use in conditions where growth fractions and repopulation rates are increasing. Hypoxia does modify α, but since the hypoxic zone cells are not repopulating there is no need to estimate shifts in mean α due to a changing hypoxic fraction. *Krepop* can change due to variation in *T_eff_* or the α radiosensitivity parameter. For changes in the latter between different tumours, modified *Krepop* values can be simply found from its defining equation. For example, for a *Krepop* value estimated using α=0.3 Gy^-1^, but with an α value of *x* Gy^-1^, then Equation 8 applies:

(8) $$Krepop=\frac{0.3\;0.693}{x\;T_{eff}},$$

so that if α changes from 0.3 to be 0.4 Gy^-1^, then the *Krepop* value will reduce by 25%, which is considerable. **Figure 6** shows the change in *Krepop* with treatment duration for different values of *T_D_*. Even if α and β are constant during treatment, the mean α/β ratio will change due to reoxygenation. For example, in the squamous cell cancer example, in **Figure 1**, the initial α/β (allowing for hypoxia) is 9.17 Gy but it reduces to 8.77 Gy at 80 days with reoxygenation, so that the present model does inherently include a change in α/β. Future modellers may extend the model to include a relationship between *T_eff_* and radiosensitivities, but this would be complex and beyond the scope of the present article.

The confidence limits of the estimated mean *Krepop* values in published data can be interpreted as representing the variation in *Krepop* at different times, where the data includes individual patients with tumours which had different volume doubling times before treatment commenced and had different overall treatment durations. Such limits could also act as a guide as to the selection of *Krepop* values according to the expected treatment duration after an interruption and its correction.

Renewed interest in treatment interruptions, their radiobiological significance and means of correction occurred during the covid pandemic (3, 4). The use of hypo-fractionated schedules which deliver radiotherapy in much shorter times can, with interruptions, lead to longer times where corrections can be considered worthwhile to maintain the target BED providing that the normal tissue BED can accommodate an increase in dose within the usual tolerance constraints. The use of variable *Krepop* values could be difficult to apply, since the standard values are averages and apply only for times greater than the apparent onset of AR, but the suggested approach taken in **Appendix C** could be considered.

The *Krepop* values given above should be treated as a guide to the likely range of practical values and are probably not necessary for long treatment durations where the existing data should be sufficient, but consideration should be given to the use of the smaller *Krepop* values in the case of treatment interruptions occurring at relatively short times in hypofractionated treatments.

The present author has noted that many research scientists and clinicians are unaware that cell proliferation rates are faster in many normal tissue systems than in cancers, with a poor appreciation of the subtleties of cell kinetics and the important role of the cell loss factor. There needs to be a renaissance of interest in the subject.

## Conclusions

The model presented provides important insights into the dynamic relationships between tumour hypoxia, reoxygenation, radiosensitivity and eventual proliferation, in a time sequence governed by the pretreatment volume doubling time (*T_D_*) which is itself related to the cell loss factor (*φ*) and its own rate of change. The unmasking of accelerated repopulation can only occur after sufficient reduction in *φ*, encouraged by reoxygenation. Previously there has been excessive emphasis on proliferation markers in isolation and which, although relevant, require the φ parameter and its time-related changes to be known. The role of *T_D_* may be paramount in this respect. Since the present model is deterministic, future versions of the model might include stochastic parameter heterogeneity effects. Further progress might follow linkage of genetic mutation data to the important cell kinetic and radiosensitivity parameters. The overall conclusion is also that *Krepop* values probably vary with duration of treatment, which has some implications for unintended treatment interruption corrections, especially in the case of hypo-fractionated schedules.

## Data Availability

The original contributions presented in the study are included in the article/supplementary material. Further inquiries can be directed to the corresponding author.

## Appendix A

*A brief summary of essential cell kinetics*

Exponential tumour volume growth, for small sized tumours, can be expressed as in Equation A1

(A1)
$$v_{t}=v_{0}\;e^{k_{G}t},$$

*v_0_* being the initial volume, *v_t_* the volume at time *t* and *k_G_* is the growth rate constant during exponential growth, which can also be expressed as in Equation A2

(A2)
$$k_{G}=k_{B}-k_{L},$$

where *k_B_* is the cell birth rate of new cells and *k_L_* is the cell loss rate.

The cell loss factor (φ) can be expressed in two ways (which are mathematically equivalent), namely as in Equation A3

(A3)
$$\varphi =\frac{k_{L}}{k_{B}},\text{ and }\varphi =\left(1-\frac{T_{pot}}{T_{D}}\right),$$

where *T_pot_* is the potential doubling time and *T_D_* is the volume doubling time, which is itself rated to *k_G_* by the relationship as in Equation A4

(A4)
$$T_{D}=\frac{0.693}{k_{G}}.\left(\text{or }T_{D}=\frac{0.693}{k_{B}-k_{L}}\right)$$

The various doubling times are related as follows:

T_C_ is the time required to traverse the cell cycle (and is around 2 days in most human tumour cells). It is modified by the growth fraction (GF) which is the fraction of cells undergoing the cell cycle, so that a *T_C_* of 2 days is modified to be a *T_pot_* of 4 days if GF is 50%, since $T_{pot}=\frac{T_{C}}{GF}$. *T_pot_* is probably a good indicator of the proliferative status of a tumour at initiation of treatment. From the second definition of φ given above, it follows that Equation A5 applies:

(A5)
$$T_{D}=\frac{T_{pot}}{\left(1-\varphi \right)}.$$

It also follows from Equation A2 that the half-times of each process will be linked as in Equation A6

(A6)
$$\frac{0.693}{T_{D}}=\frac{0.693}{T_{pot}}-\frac{0.693}{T_{loss}},\text{ or }\frac{1}{T_{D}}=\frac{1}{T_{pot}}-\frac{1}{T_{loss}},$$

where *T_loss_* is the half-time of tumour cell death.

*T_D_* can be expressed in a form which, although more complex, illustrates its dependence on the fundamental cell kinetic parameters, as in Equation A7

(A7)
$$T_{D}=\frac{T_{C}}{GF.\;\left(1-\varphi \right)}.$$

For a more complete exposition of cell kinetics and the techniques used to develop this branch of biophysics, there are two important textbooks by Steel (1977) (39) and Aherne, Camplejohn and Wright (1976) (40),, which should be consulted. The latter gives more prominence to the stathmokinetic method utilising the metaphase arrest obtained with Vinca alkaloid drugs to estimate cell birth rates (*k_B_*) and so the *T_pot_*.

The *T_eff_* concept can be understood as the operative doubling time of tumour cells during radiotherapy, which may vary between the initial *T_D_* value and, with increasing treatment duration, to approach *T_pot_*, and further towards *T_C_*.

Appendix B

The actual Mathematica programme is given below in text form, where the following replacement symbols, designed to avoid confusion during programming, are used:

*T_C_* is tc, *T_pot_* is tpot, *Krepop* is kprol, h is the hypoxic fraction, *k_L_*_is_ kloss, f is the interfraction interval, total dose is td, number of fractions are n; qa, qb are the respective hypoxia reduction factors for α and β respectively, α is a, β is b, φ_0_ is phi0, and φ_t_ is phi, z is the rate constant for the reduction in φ.

sfox and sfhypox are the respective surviving fractions in oxic and hypoxic compartments, and ncts is the number of pre-treatment clonogens.

Lists are named by use of the above symbols followed by list, e.g. kplist is the list of kprol (i.e. K values).

Note that TCP is estimated as probability values between 0 and 1 and are then converted to percentages.

The following programme is for the Squamous cell carcinoma example.

***Programme***

Clear[ncts, t, w, f, h, tcp, tcplist, kp, kplist, sfox,noxlist, nhyplist, hlist, tlist, kloss, z, a, b, phi0, phi, qa, qb];

ncts=5 10^8; phi0 = 0.9; philist={phi}; gf=0.5;tc=1.9; w=tc/gf; z=1/(tpot/(1-phi0)); h0 = 0.15; n=1;d=2; f=7/5; t=0; a=0.36; b=0.036; qa=1.75; qb=3.5;

sfox=Exp[-a d-b d^2];

sfhypox=Exp[- a d/qa-b/qb d^2];r=f (1-sfhypox)/(1-sfox) 0.693/tpot;

kloss=phi0 0.693/tpot;

tlist={0}; hlist={h0}; t=0;

nox=(1-h) ncts; nhyp=h ncts; nhyp=ncts h0 Exp[-a/qa d-b/qb d^2]; nox=ncts (1-h0) Exp[-a d-b d^2];

teff=tpot/(1-phi0);

nlist={};noxlist={nox}; nhyplist={nhyp};

tefflist={teff}; tcplist={0}; td=n d; tdlist={td}; nhyp=nhyp Exp[-a/qa d-b/qb d^2]; nox= nox Exp[-a d-b d^2]; n=2; nlist=Append[nlist, n];

h=nhyp/(nox+nhyp); kplist={};kprol=0.693/(a teff);

kplist=Append[kplist, kprol];

Do[Label[ejj];n=n+1; nlist=Append[nlist, n];

t=f(n-1); tlist=Append[tlist, t]; nhyp=nhyp Exp[-a/qa d-b/qb d^2];

If[t<3 tc, kloss=f phi0 0.693/w, kloss=f phi0 Exp[-z (t-3 tc)] 0.693/tc];

nox= nox Exp[-a d-b d^2 + 0.693 f/teff]; nox=nox+ kloss nhyp;

nhyp=nhyp- kloss nhyp; h=nhyp/(nox+nhyp); hlist=Append[hlist, h];

If[t<3 tc, teff=teff, teff=tpot/(1-(phi0 Exp[- z (t- 3 tc)]))- (tpot-tc)];

tefflist=Append[tefflist, teff]; kprol=0.693/(a teff);

noxlist=Append[noxlist, nox]; nhyplist=Append[nhyplist, nhyp]; td=n d; tdlist=Append[tdlist, td]; kplist=0.693/(tefflist a);

tcp=If[nox>8, 0, 100Exp[-(nox+nhyp)]]; tcplist=Append[tcplist, tcp];

If[t<80, Goto[ejj], Break[]]];

cordsteff=Transpose[{N[tlist], tefflist}];

cordsh=Transpose[{N[tlist], 100 hlist}];

cordstcp=Transpose[{N[tlist], tcplist}];

cordstd=Transpose[{N[tlist], tdlist}];

cordskp=Transpose[{N[tlist], 100 kplist}];

ListPlot[{cordsteff,cordstcp, cordstd, cordsh, cordskp}, PlotRange->All, Joined->True, Frame->True, FrameLabel->{“Fractionated Treatment Duration (days)”,”Estimated Responses (%)”},BaseStyle->{14,FontFamily->“HelveticaBold”},

PlotLabel->“Squamous Cell Cancer”, PlotStyle->{{Blue, PointSize[0.01]},{Black,PointSize[0.007]}, {Gray, PointSize[0.006]}, {Brown,PointSize[0.02]},{Red,PointSize[0.007]}, FrameTicks->Automatic},Epilog->{FontSize->9,{Text[“TCP (black)”,{32, 70}]},{Text[“h(brown)”,{50, 10}]},{Text[“Total Dose(Gy)(grey)”,{60, 110}]},{Text[“Krepop(red)”,{60, 60}]},{Text[“Teff(blue)”,{10,40}]}}]

Appendix C

The following theoretical example will illustrate a method for inclusion of a variable K value in treatment interruption compensatory dose estimations. To ease understanding the following notation and symbolism is perhaps helpful.

The intended net tumour BED (BED_int_), allowing for repopulation, in an uninterrupted treatment is

*BEDint* = Radiation BED – BED_repop_ in time T,

Or in symbolic form as in Equation C1

(C1)
BEDint=N d1(1+d1(αβ)tum)−g(T),

Where N and d_1_ are number of fractions of dose d1, and *g(T)* is also a function of *K, T_eff_ and α*, the term being the BED equivalent of repopulation in the intended treatment time T.

In the context of a treatment interruption at a time T_A_, the net delivered BED can be expressed as:

BED = Prescribed BED - BED_repop_ in time T_A_

or in symbolic form as in Equation C2

(C2)
BED=NA d1(1+d1 (αβ)tum)−g(TA)

For the interrupted treatment and its compensation, which includes an extension of treatment time to a time T_F_, then the compensated tumour BED (BED_comp_) will be

BED_comp_ = radiationBED_A_ delivered in time T_A_ + radiationBED_C_ delivered between times T_A_ and T_C_ – BED_repop_ in time T – BED_repop_ in time T_F_ – T,

Or in symbolic form, where *N_A_* is the number before the interruption, and *N_C_* is the number of fractions of dose *d_2_* given after the interruption, Equation C3 then applies:

(C3)
BEDcomp=NA d1(1+d1(αβ)tum)+NC d2(1+d2(αβ)tum)−g(TA)−g(T−TA)−g(TC−T),

and where the three time-functions can be simplified as $g\left(T\right)-g\;\left(T_{C}-T\right)$.

In order to estimate the fractional compensatory dose in BED_C_ to provide complete restoration of tumour BED, Equations C1 and C3 are equated, which eliminates the two *g(T)* functions within the resultant equation, and now the time function is given in its BED form, so that Equation C4 then applies:

(C4)
N d1(1+d1(αβ)tum)=NA d1(1+d1(αβ)tum)+NC d2(1+d2(αβ)tum)−mean Krepop(TF−T)

If N_C_ is decided, the dose d_2_ can be estimated. The mean *Krepop* value over the relevant time $\left(T_{F}-T\right)$ must include the mean T_eff_ value which can either be assessed visually or found by integration of the *φ* function to provide the mean *φ*, as in Equation C5

(C5)
$$\bar{\varphi }=\frac{\int_{T}^{T_{F}}\varphi_{o}e^{-z\left(T-3T_{C\;}\right)}}{\left(T_{F}-T\right)}\cdot dT=\varphi_{0}\frac{e^{-z\left(T+T_{F}+3T_{C\;}\right)\left(e^{zT}+\;e^{zT_{F}}\right)}}{z\left(T_{F}-T\right)}$$

which and can estimate the mean *T_eff_* from the relationship, Equation C6

(C6)
$$\bar{T_{eff}}=\frac{T_{pot}}{\left(1-\bar{\varphi }\right)}-\left(T_{pot}-T_{C\;}\right).$$

The mean *Krepop* is then given by

$$\frac{0.693}{\alpha \times \bar{T_{eff}}}$$

The full restoration of the intended BED is then found and will in most cases exceed the normal tissue tolerance (*BED_TOL_*), so a compromise is usually required and achieved by successive iterations of Equation C4 to satisfy Equation C7

(C7)
NA d1 x(1+d1 x(αβ)OAR)+NC d2 x(1+d2 x(αβ)OAR)≤BEDTOL

using (αβ)OAR values for the organ at risk and also including the degree of normal tissue sparing (*x*).

If, for example, a carcinoma has an interruption which extends a 49 day treatment course to be 56 days, then the mean value of *Krepop* should be that estimated for the 8^th^ week of treatment. If a hypo-fractionated treatment intended to be completed in 12 days is interrupted but extended to be 24 days, then the operative *K* should be that estimated between those two times by taking an averaged value. It is beyond the scope of the present article to provide long and detailed worked examples, but these may be made available in future publications.
